# Structural insights into the substrate specificity of two esterases from the thermophilic *Rhizomucor miehei*

**DOI:** 10.1194/jlr.M060673

**Published:** 2015-08

**Authors:** Shaoqing Yang, Zhen Qin, Xiaojie Duan, Qiaojuan Yan, Zhengqiang Jiang

**Affiliations:** * College of Food Science and Nutritional Engineering, The Research and Innovation Center of Food Nutrition and Human Health (Beijing), China Agricultural University, Beijing 100083, China; † College of Engineering, China Agricultural University, Beijing 100083, China

**Keywords:** crystal structure, hormone-sensitive lipase, substrate-binding pocket, structure-function relationship

## Abstract

Two hormone-sensitive lipase (HSL) family esterases (*Rm*EstA and *Rm*EstB) from the thermophilic fungus *Rhizomucor miehei*, exhibiting distinct substrate specificity, have been recently reported to show great potential in industrial applications. In this study, the crystal structures of *Rm*EstA and *Rm*EstB were determined at 2.15 Å and 2.43 Å resolutions, respectively. The structures of *Rm*EstA and *Rm*EstB showed two distinctive domains, a catalytic domain and a cap domain, with the classical α/β-hydrolase fold. Catalytic triads consisting of residues Ser161, Asp262, and His292 in *Rm*EstA, and Ser164, Asp261, and His291 in *Rm*EstB were found in the respective canonical positions. Structural comparison of *Rm*EstA and *Rm*EstB revealed that their distinct substrate specificity might be attributed to their different substrate-binding pockets. The aromatic amino acids Phe222 and Trp92, located in the center of the substrate-binding pocket of *Rm*EstB, blocked this pocket, thus narrowing its catalytic range for substrates (C2–C8). Two mutants (F222A and W92F in *Rm*EstB) showing higher catalytic activity toward long-chain substrates further confirmed the hypothesized interference. This is the first report of HSL family esterase structures from filamentous fungi.jlr The information on structure-function relationships could open important avenues of exploration for further industrial applications of esterases.

Esterases (EC 3.1.1.1) are a general class of carboxylic ester hydrolases, which catalyze the cleavage and formation of ester bonds (1). They exhibit maximum activity toward water-soluble or emulsified esters of short-chain carboxylic acids (less than 10 carbon atoms), a property that distinguishes them from lipases. They are widely used in the food, perfume, cosmetic, chemical, agricultural, and pharmaceutical industries, owing to their unique properties: tolerance to organic solvents, substrate specificity, and stereoselectivity (2–4). However, there are still some barriers to the use of esterases in industrial applications, such as low production yield, limited pH and thermal stability, and poor performance in organic solvents (5, 6). For certain industrial applications, functional modification of the enzymes based on rational protein design is necessary to enhance their catalytic efficiency, substrate specificity, enantioselectivity, and thermostability (7–10). Better knowledge of their structure and function will facilitate rational design.

Lipolytic enzymes have been classified into four blocks (C, L, H, and X) on the basis of similarities in amino acid sequence and the presence of several conserved motifs, as described in the ESTHER database (11). Block H is composed of plant carboxylesterase and hormone-sensitive lipase (HSL) families. The HSL family consists of esterases and lipases that are distributed across diverse organisms, including animals, plants, and microorganisms (1); they share high sequence similarity with mammalian HSL (12). HSL family esterases exhibit broad substrate spectrum, with a significant lipolytic activity on triacylglycerol, diacylglycerol, monoacylglycerol, and cholesteryl ester. Some of them playing a role in lipid catabolism or detoxification have been proposed (13, 14). To date, a number of HSL family esterases have been identified and biochemically characterized from various microorganisms and metagenomic libraries (1, 15, 16). Moreover, several of them have been structurally elucidated, including *Pc*Est from *Pyrobaculum calidifontis* [Protein Data Bank (PDB) PDB: 2YH2], *Af*Est from *Archaeoglobus fulgidus* (PDB: 1JJI), *St*Est from *Sulfolobus tokodaii* (PDB: 3AIK), *Aa*Est from *Alicyclobacillus acidocaldarius* (PDB: 1EVQ), *Bs*Est from *Bacillus subtilis* (PDB: 1JKM), and EstEl (PDB: 2C7B) and EstE7 (PDB: 3DNM) from metagenomic libraries. However, there have been no structural descriptions of HSL family esterase from a filamentous fungus.

HSL family esterases share a characteristic α/β-hydrolase fold, which is composed of a central β-sheet surrounded on both sides by α-helices, serving as a stable protein core. The amino acid substitutions, loop insertions, and deletions occurring in the central cores during evolution have led to enzymes with diverse catalytic functions (17). The catalytic mechanism of α/β hydrolase is based on a catalytic triad made up of a nucleophile (Ser), an acid (Asp or Glu), and a His. The central nucleophile is located within a conserved G-X-S-X-G motif in the “nucleophile elbow” (18). Though the catalytic mechanism is nearly identical in all α/β hydrolases, their substrate specificities are very different. The reasons for this phenomenon have not been elucidated at the structural level.

We recently characterized two novel HSL family esterases from the thermophilic fungus *Rhizomucor miehei*: *Rm*EstA and *Rm*EstB (15, 16). The physiological substrates of *Rm*EstA and *Rm*EstB detected in our studies have been found to be short-chain triacylglycerol, linalyl acetate, and butyl butyrate. Both of these enzymes belong to the α/β hydrolases and exhibit distinct substrate specificities: *Rm*EstA shows highest activity toward longer-chain esters, whereas *Rm*EstB favors hydrolysis of shorter-chain esters. Here, to decipher the structural basis of their different substrate specificity, we report the crystal structures of the two esterases and detail their structural differences. This work is a first attempt to characterize HSL family esterases from a filamentous fungus at the structural level to gain insights into their structure–function relationships.

## MATERIALS AND METHODS

### Gene cloning, site-directed mutagenesis, protein expression, and purification

The two recombinant esterases (*Rm*EstA and *Rm*EstB) from *R. miehei* CAU432 were cloned, expressed, and purified according to protocols described in our previous studies (15, 16). Site-directed mutations of *Rm*EstB were generated with the Fast Mutagenesis System site-directed mutagenesis kit (TransGen Biotech, China). For the mutation W92F, the primers were 5′-TTCCATGGTGGAGGTTTTGTTGTTGGCAG-3′ (forward) and 5′-AAAACCTCCACCATGGAAGAAGACGATAGG-3′ (reverse). For the mutation F222A, the primers were 5′-CAAGCTGATGGTTTGGTTTGCTGATCACTATAT-3′ (forward) and 5′-GCAAACCAAACCATCA­G­CTTGCGTGTGAGATAAT-3′ (reverse). The variants were expressed and purified as described previously (16). The purity of the proteins was checked by SDS-PAGE. Prior to crystallization, the protein samples were moved to 20 mM Tris-HCl buffer pH 8.0 containing 100 mM NaCl and concentrated to 15 mg/ml.

### Enzyme assays and protein determination

Esterase activity was determined as described by Gutiérrez-Fernández et al. (19) using *p*-nitrophenyl acetate (*p*NPA) as the substrate with minor modifications. Briefly, 50 μl of suitably diluted enzyme solution was prepared in 400 μl of 50 mM Tris-HCl buffer pH 7.5, and after preheating for 2 min, 50 μl of 20 mM *p*NPA substrate (in pure isopropanol) was added. The mixture was incubated at 50°C for 10 min. The reaction was stopped by adding 500 μl of 300 mM phosphate buffer pH 7.0 containing 5% (w/v) SDS. The released *p*-nitrophenol (*p*NP) was quantified by measuring the absorbance at 410 nm. One unit of enzyme activity was defined as the amount of enzyme required to liberate 1 μmol *p*NP per minute under the above assay conditions. Protein concentration was measured by the Lowry method using BSA as the standard. Specific activity was expressed in units per milligram protein.

### Protein crystallization and X-ray data collection

Proteins were crystallized by the sitting-drop vapor diffusion method at 293 K by mixing 1 µl protein solution with an equal volume of reservoir solution. Crystals of *Rm*EstA were obtained with a reservoir solution containing 25% (w/v) polyethylene glycol (PEG) 3350 and 0.2 M (NH_4_)_2_SO_4_ in 0.1 M MES buffer pH 6.0. The *Rm*EstA crystals were observed 2 days later. Crystals of *Rm*EstB were obtained with a reservoir solution containing 20% (w/v) PEG4000 and 10% (v/v) 2-propanol in 0.1 M HEPES buffer pH 7.5. The *Rm*EstB crystals were observed after 7 days.

For X-ray diffraction experiments, each crystal was fished from the crystallization drop using a nylon loop (Hampton Research), soaked briefly in a cryoprotectant solution (the crystallization solution supplemented with 20% v/v glycerol), and then flash-cooled in liquid nitrogen at 70 K. X-ray diffraction data of *Rm*EstA and *Rm*EstB were collected from single crystals at beamline 3W1A at the Beijing Synchrotron Radiation Facility (BSRF) and beamline BL-17U at the Shanghai Synchrotron Research Facility (SSRF), respectively. All diffraction data were indexed, integrated, and scaled using the program HKL-2000 (20).

### Structure determination and refinement

The structure of *Rm*EstB was determined by the molecular replacement (MR) method using the structure of PDB entry 1JJI (*Archaeoglobus fulgidus* carboxylesterase) as the search model. The structure of *Rm*EstA was solved by MR using the refined structure of *Rm*EstB as the search model. The structural models were built and refined using the Phenix (21) and Coot (22) programs. *R* values for all data were reduced by several cycles of simulated annealing, minimization, and *B*-factor refinement using Phenix.refine followed by manual model rebuilding. The final models were analyzed and validated with MolProbity (23). Structural homologs of esterases were identified in the DALI server (24). Structural superpositions and RMSD (root-mean-square deviation) calculation were performed with the LSQMAN program (25). Secondary-structure elements were identified by the DSSP (*Define Secondary Structure of Proteins*) program (26). Figures were prepared with PyMOL (27). Sequence alignment was created by ClustalW (28). Data collection and refinement statistics are given in **Table 1**.

**TABLE 1.** Data collection and refinement statistics

Data Collection Statistics	*Rm*EstA	*Rm*EstB
Wavelength (Å)	0.9791	0.9792
Resolution range (Å)	31.85–2.41 (2.50–2.41)	46.65–2.27 (2.35–2.27)
Space group	*P*1	*C*121
Unit cell parameters		
* a*, *b*, *c* (Å)	56.69, 64.52, 64.79	151.13, 49.68, 183.21
Unique reflections	26,749 (2,373)	55,910 (4,188)
Completeness (%)	97.31 (86.07)	94.49 (71.8)
*R*_merge_ (%)*^a^*	5.1 (35.6)	7.5 (46.6)
*I*/σ(*I*)	14.22 (3.54)	15.08 (8.28)
Wilson *B*-factor	34.29	23.20
Refinement statistics		
Resolution range (Å)	2.41	2.27
* R*_work_ (%)/*R*_free_ (%)*^b^*	17.75/22.56	18.84/23.59
No. atoms	5,143	10,735
No. residues	640	1,294
No. water molecules	269	529
RMSD		
Bond lengths (Å)	0.011	0.010
Bond angles (°)	1.30	1.24
Ramachandran favored (%)	96	96
Ramachandran outliers (%)	0.31	0.16
Average *B*-factor (Å^2^)	31.90	28.40
PDB code	4WY5	4WY8

Values in parenthesis are from the last resolution shell.

a *R*_merge_ = Σ*_hkl_*Σ*_i_*[*I_i_*(*hkl*) − *I*(*hkl*)]/Σ*_hkl_*Σ*_i_I_i_*(*hkl*), where *I_i_*(*hkl*) is the *i*th observation of reflection *hkl* and* I*(*hkl*) is the weighted average intensity for all observations *i* of reflection *hkl*.

b *R*_merge_ = Σ*_hkl_*Σ*_i_*[*I_i_*(*hkl*) − *I*(*hkl*)]/Σ*_hkl_*Σ*_i_I_i_*(*hkl*); 95% and 5% of reflections were used for *R*_work_ and *R*_free_, respectively.

### Substrate-specificity analysis

Substrate specificity of *Rm*EstB mutants were investigated according to standard enzyme assay in 50 mM Tris-HCl buffer pH 7.5 at 50°C using different *p*NP esters as the substrates, including *p*NPA, *p*-nitrophenyl butyrate (*p*NPB), *p*-nitrophenyl hexanoate (*p*NPH), *p*-nitrophenyl caprylate (*p*NPC), *p*-nitrophenyl decanoate (*p*NPD), *p*-nitrophenyl laurate (*p*NPL), *p*-nitrophenyl myristate (*p*NPM) and *p*-nitrophenyl palmitate (*p*NPP). One unit of enzyme activity was defined as the amount of enzyme required to release 1.0 μmol of *p*NP per minute under the above assay conditions.

### Accession numbers

The atomic coordinates and structural factors for crystal structures of *Rm*EstA and *Rm*EstB were deposited in the PDB under accession numbers 4WY5 and 4WY8, respectively.

## RESULTS

### Overall structures of *Rm*EstA and *Rm*EstB

**Fig. 1.**
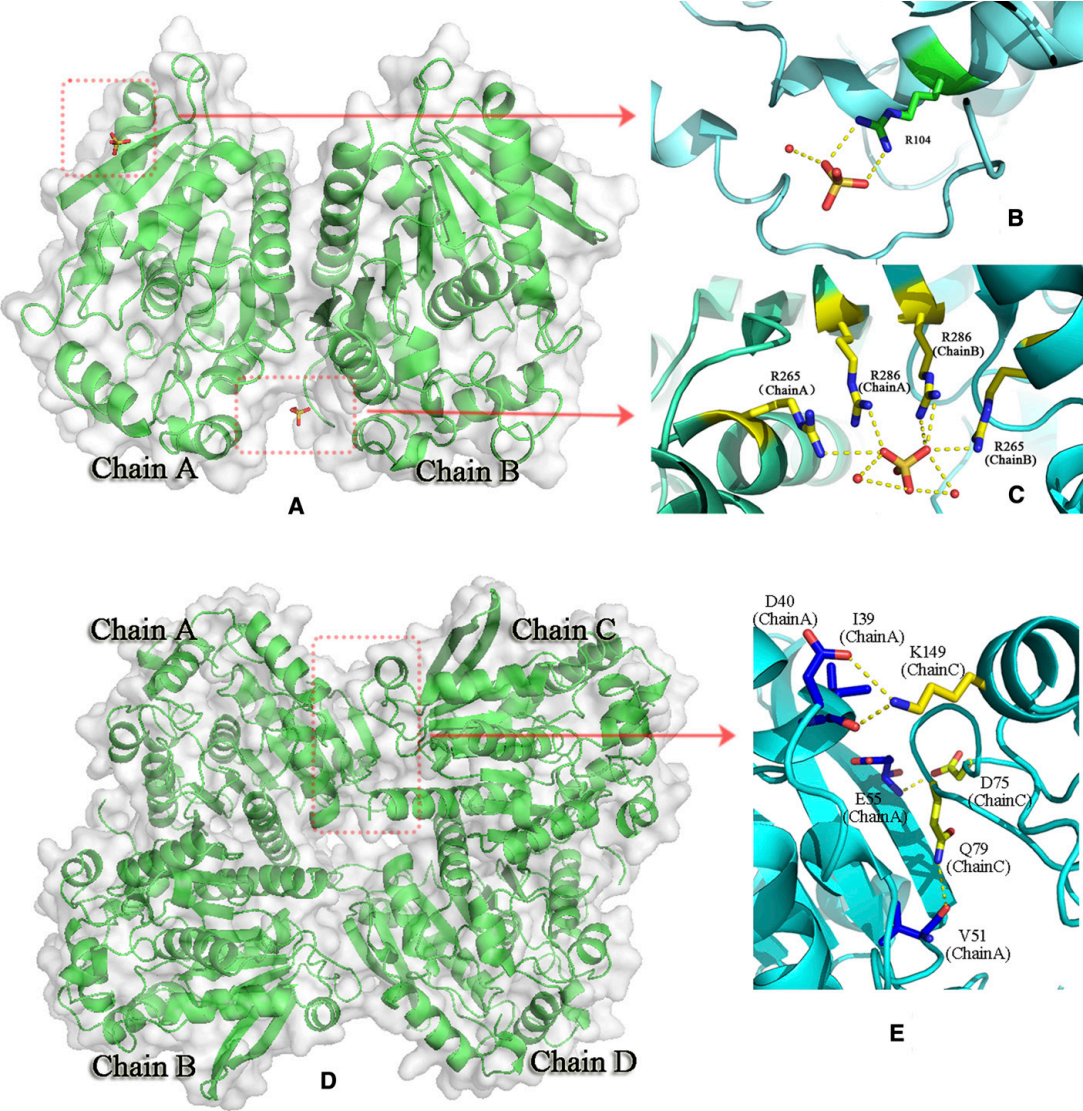
Three-dimensional structures and molecular surfaces of *Rm*EstA and *Rm*EstB. A: The two molecules of the monomer present in the asymmetric unit of *Rm*EstA. B: Each monomer of the *Rm*EstA complex with a sulfate radical, which forms a hydrogen bond with residue Arg104 and water. C: Sulfate radical in the crevice of two monomers. This ligand combines, via hydrogen bonding, with residues Arg265 and Arg286 from each monomer. D: Four molecules of monomer present in the asymmetric unit of *Rm*EstB. The four subunits are shown in different colors. E: The associated hydrogen bonding network formed between chain A and chain C.

The crystal structures of *Rm*EstA and *Rm*EstB were determined at 2.41 Å and 2.27 Å resolutions, respectively. The crystallographic statistics for data collection and structure refinement are summarized in Table 1. The triclinic space group of *Rm*EstA was *P*1 with two monomers in the asymmetric unit (**Fig. 1A**). Amino acid residues 1–3 of *Rm*EstA and the uncleaved C-terminal His-tag were not visible on the electron-density map. In the asymmetric unit, *Rm*EstA formed a dimer in complex with three sulfates, and two of the sulfates interacted with two monomers via Arg104 (Fig. 1B). Note that the third single sulfate was found in the crevice of two monomers, with four Arg residues from two monomers forming an arched area (Fig. 1C). The monoclinic space group of *Rm*EstB was *C*121 with four molecules in the asymmetric unit. The four monomers were arranged as two canonical dimers to further form a tetramer via hydrogen bonding network (Fig. 1D). Four hydrogen bonds were involved in the formation of the tetramer: Ile39 and Asp40 of chain A were directly hydrogen bonded to Lys149 of chain C, Glu55 of chain A was directly hydrogen bonded to Asp75 of chain C, and Val51 of chain A was directly hydrogen bonded to Gln79 of chain C (Fig. 1E).

**Fig. 2.**
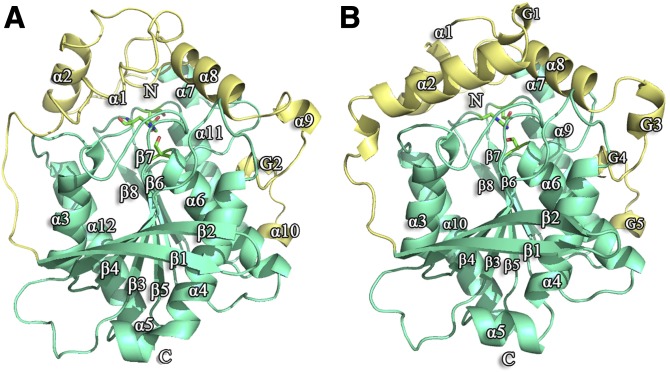
Overall folds of *Rm*EstA and *Rm*EstB monomers. Ribbon diagrams of the structures of *Rm*EstA (A) and *Rm*EstB (B) showing the classical α/β-hydrolase fold. The catalytic domains are shown in cyan, and the cap domains in yellow. The active sites of *Rm*EstA and *Rm*EstB are shown in stick models in green.

The overall structure of the *Rm*EstA monomer was analogous to that of the *Rm*EstB monomer (**Fig. 2**). The structures of *Rm*EstA and *Rm*EstB could be divided into two domains: a catalytic domain (residues 51–191 and 253–322 for *Rm*EstA, residues 51–191 and 253–322 for *Rm*EstB) and a cap domain (residues 4–51 and 207–247 for *Rm*EstA, residues 3–51 and 206–247 for *Rm*EstB). The catalytic domains had the canonical architecture of an α/β-hydrolase fold protein consisting of a central β-sheet of eight mostly parallel strands surrounded by α-helices (Fig. 2). The core β-sheets of each monomer were related by 2-fold symmetry to form an extended intermolecular 16-stranded β-sheet. The central β-sheet displayed a left-handed superhelical twist, with β1 and β8 strands crossing each other at an angle of ∼120° (Fig. 2).

### Structural comparison of *Rm*EstA and *Rm*EstB with other esterases

The analysis of structural similarity carried out with DALI search suggested high structural similarity of both *Rm*EstA and *Rm*EstB with other reported esterases. *Rm*EstA exhibited high structural similarity with the esterases from *P. calidifontis* (*Pc*Est, PDB code: 3ZWQ), *A. fulgidus* (*Af*Est, PDB code: 1JJI), *Salmonella typhimurum* (*St*Est, PDB code: 3GA7), and *A. acidocaldarius* (*Aa*Est, PDB code: 1EVQ), with Z-score and RMSD of C^α^ atom values of 39.7 and 1.9, 39 and 2.0, 38.8 and 2.1, and 38.2 and 2.2, respectively. *Rm*EstB displayed high structural similarity with the esterases from *P. calidifontis* (*Pc*Est, PDB code: 3ZWQ), *A. fulgidus* (*Af*Est, PDB code: 1JJI), *A. acidocaldarius* (*Aa*Est, PDB code: 1EVQ), and metagenomic library (EstE1, PDB code: 2C7B), with Z-score and RMSD of C^α^ atom values of 41.5 and 1.8, 40.5 and 1.9, 40.3 and 2.1, and 40.3 and 1.7, respectively. Note that the three-dimensional structures of *Rm*EstA and *Rm*EstB shared high similarity with those of the other members of the α/β-hydrolase fold family, though they showed low sequence identities (<40%). Superimposing *Rm*EstA/*Rm*EstB on the structures of *Pc*Est and *Af*Est revealed similar overall folds of these HSL family esterases. The structural differences were found mainly in the loop regions.

### The active sites of *Rm*EstA and *Rm*EstB

**Fig. 3.**
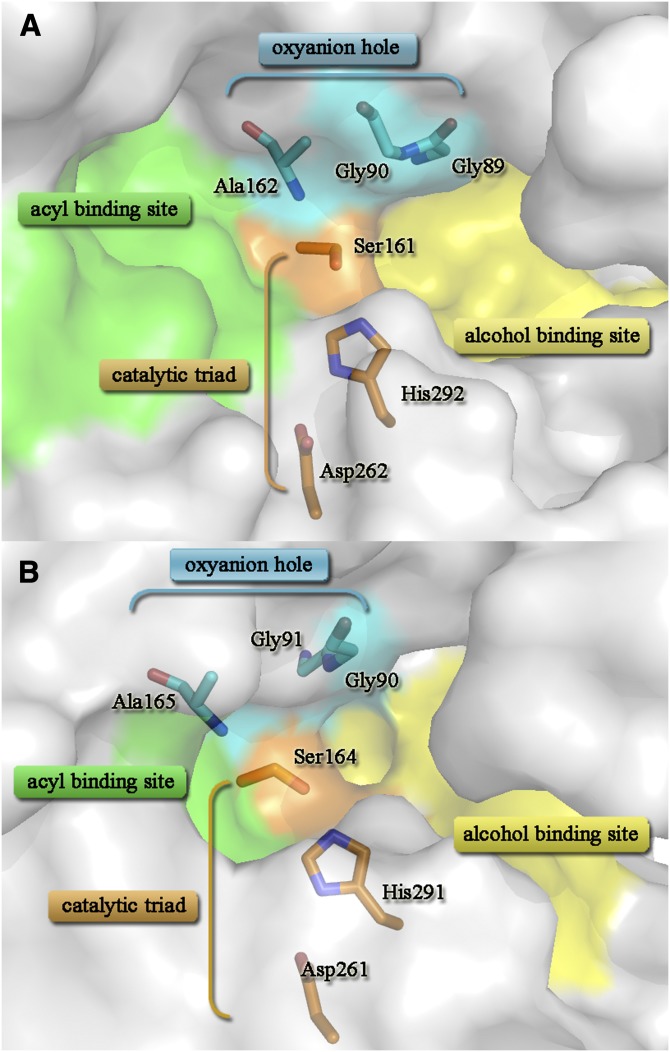
Active sites of *Rm*EstA and *Rm*EstB. The active sites of *Rm*EstA (A) and *Rm*EstB (B) are shown in surface view. The residues of the catalytic triad are shown as stick models in orange, and the residues of the oxyanion hole are shown as stick models in blue. The large parts of the acyl- and alcohol-binding site of esterases are in the green and yellow regions. The acyl binding site and alcohol binding site were identified by structural superposition according to several reported structures of HSL family esterases [PDB code: 3ZWQ (33); PDB code: 1QZ3 (38)], which means the binding sites toward the acyl part and the alcohol part of the substrate, respectively.

A classical catalytic triad consisting of Ser161 (Ser164 in *Rm*EstB) as the nucleophile, His292 (His291 in *Rm*EstB) as the proton acceptor/donor, and Asp262 (Asp261 in *Rm*EstB) as the residue stabilizing the His was identified in *Rm*EstA (*Rm*EstB) (**Fig. 3**). The key nucleophile Ser161 in *Rm*EstA (Ser164 in *Rm*EstB) was found within the conserved pentapeptide sequence Gly-X-Ser-X-Gly, which is located at the apex of the nucleophile elbow, a sharp turn connecting β5 and α6 (5). A hydrogen bond (2.7 Å in *Rm*EstA, 2.4 Å in *Rm*EstB) between the O^γ^ atom of Ser161 (Ser164 in *Rm*EstB) and the N^ε2^ atom of His292 (His291 in *Rm*EstB) stabilized the conformation of the nucleophile Ser161 in *Rm*EstA (Ser164 in *Rm*EstB). The side chains of His292 and Asp262 in *Rm*EstA were stabilized by a network of hydrogen bonds located at the carboxyl edge of β-strands 7 and 8, respectively. However, this phenomenon was not found in *Rm*EstB. The His-Gly-Gly-Gly motif (residues 87–90 in *Rm*EstA and residues 88–91 in *Rm*EstB), which is usually conserved in the HSL family, was found upstream of the active sites. The oxyanion hole was created by residues Gly89, Gly90, and Ala162 in *Rm*EstA, and by Gly90, Gly91, and Ala165 in *Rm*EstB (Fig. 3). The main-chain nitrogen atoms of the oxyanion hole donate hydrogen to the cleaved substrate (29), stabilizing the negative charges on the tetrahedral intermediates arising from the nucleophilic attack of Ser161 in *Rm*EstA or Ser164 in *Rm*EstB.

### Comparison with *Rm*EstA and *Rm*EstB

**Fig. 4.**
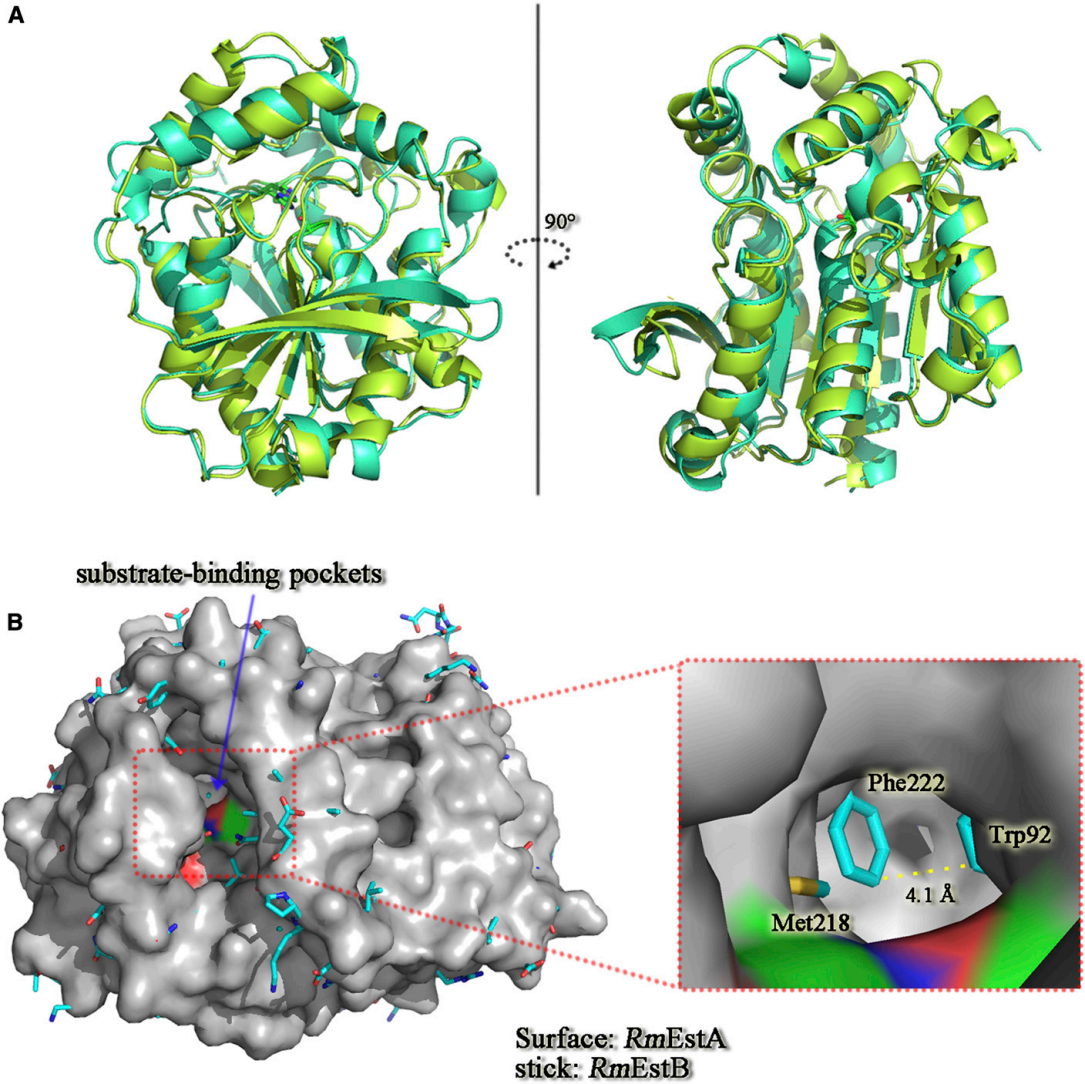
Structural comparison of *Rm*EstA and *Rm*EstB. A: Superposition of *Rm*EstA (blue) and *Rm*EstB (green) in ribbon diagrams. The overall structures of *Rm*EstA and *Rm*EstB exhibit the classical α/β-hydrolase fold, with structural differences being confined mainly to the loop regions. B: Superposition of surface of *Rm*EstA and sticks of *Rm*EstB. To display the catalytic triad molecules in *Rm*EstA, oxygen atoms are in red, nitrogen atoms are in blue, and carbon atoms are in green. *Rm*EstA and *Rm*EstB clearly have disparate substrate-binding sites. The substrate-binding channel of *Rm*EstB is closed by the aromatic residues Trp92 and Phe222.

Superposition analysis revealed high structural homogeneity between *Rm*EstA and *Rm*EstB (**Fig. 4A**). Superpositioning of *Rm*EstA onto *Rm*EstB exhibits an overall RMSD of 1.41 Å for 311 corresponding C^α^ atoms, though they shared only 46% sequence identity. The folding patterns of *Rm*EstA and *Rm*EstB presented a common core domain, where the assignment of the secondary-structure elements was almost the same. Residues 4–51 of *Rm*EstA and residues 3–51 of *Rm*EstB made up the cap domains upstream of their respective catalysis domains. It is interesting that the two cap domains shared no sequence similarity but formed similar tertiary structures. Superposition of the surface of *Rm*EstA and sticks of *Rm*EstB suggested that their most striking structural differences were localized in the substrate-binding pockets (Fig. 4B). The substrate-binding pocket of *Rm*EstB extended ∼11 Å from the protein surface to the catalytic residue Ser164. This deep hydrophobic cleft was funnel-shaped and surrounded by four α-helices (α1, α2, α6, and α8) and the loop regions (His88–Gly91 and Ile290–Ala297). The substrate-binding pocket of *Rm*EstA was a channel running through the whole protein and the entrance to this channel was surrounded by five α-helices (α1, α2, α6, α8, and α9), the 3^10^-helix G2 (His197–Lys199) and the loop regions (His87–Gly90 and Ile291–Ala298) (Fig. 4B).

### Substrate specificities

The structural differences between the substrate-binding pockets of the two esterases (*Rm*EstA and *Rm*EstB) might contribute to the difference in carbon chain lengths of the ester substrates (Fig. 4). Two aromatic amino acids (Phe222 and Trp92), located in the center of the substrate-binding pocket of *Rm*EstB, might block this pocket and narrow the substrate specificity of *Rm*EstB. To validate this speculation, two mutants (*Rm*EstB-F222A and *Rm*EstB-W92F) were designed, and the substrate specificities of two mutants were determined. Compared with that of the wild-type enzyme, the specific activity of the mutant *Rm*EstB-F222A toward C2 was slightly decreased. However, the specific activity of *Rm*EstB-F222A toward esters with relatively longer chains, such as C4 and C6, increased significantly by 1.65- and 1.4-fold, respectively (**Table 2**). The mutant *Rm*EstB-W92F showed similar results: the specific activities of *Rm*EstB-W92F toward C4 and C6 increased by 1.33- and 1.11-fold, respectively, whereas that toward C2 remained almost unchanged (Table 2).

**TABLE 2.** Substrate specificities of *Rm*EstA, *Rm*EstB, *Rm*EstB-F222A, and *Rm*EstB-W92F

	*Rm*EstA*^a^*		*Rm*EstB*^a^*		*Rm*EstB-F222A		*Rm*EstB-W92F	
Substrate	Specific Activity (U/mg)	Relative Activity (%)	Specific Activity (U/mg)	Relative Activity (%)	Specific Activity (U/mg)	Relative Activity (%)	Specific Activity (U/mg)	Relative Activity (%)
*p*NPA (C2)	370 ± 5.2	24	255 ± 5.4	100	223.8 ± 4.5*^b^*	100	226.8 ± 6.7*^b^*	100
*p*NPB (C4)	1,250 ± 24	84	92 ± 2.6	36	132.6 ± 3.5*^b^*	59.4	106.2 ± 3.3*^bc^*	48
*p*NPH (C6)	1,480 ± 36	100	19.1 ± 0.4	7.5	23.4 ± 1.5*^b^*	10.5	18.6 ± 0.6*^c^*	8.3
*p*NPC (C8)	850 ± 11	57	10.5 ± 0.4	4.1	6 ± 0.2*^b^*	2.6	13.8 ± 0.4*^bc^*	6.4
*p*NPD (C10)	380 ± 6.9	25	3.5 ± 0.1	1.4	4.2 ± 0.3*^b^*	1.9	10.2 ± 0.2*^bc^*	4.5
*p*NPL (C12)	150 ± 4.2	10	1.3 ± 0.03	0.5	1.68 ± 0.06*^b^*	0.7	1.8 ± 0.05*^bc^*	1
*p*NPM (C14)	20 ± 0.8	1.4	0.6 ± 0.01	0.2	0.18 ± 0.04*^b^*	0.1	0.6 ± 0.01*^c^*	0.3
*p*NPP (C16)	6 ± 0.2	0.4	0.05 ± 0	0.01	NA*^bd^*	0	NA*^b^*	0

Data represent the means ± SD of three independent experiments (n = 3).

a Substrate specificities of *Rm*EstA and *Rm*EstB have been reported in the previous studies (15, 16).

b *P* < 0.05 compared with *Rm*EstB (one-way ANOVA).

c *P* < 0.05 compared with *Rm*EstB-F222A (one-way ANOVA).

d No activity detected.

## DISCUSSION

The α/β-hydrolase superfamily is one of the largest enzyme superfamilies recognized to date and ubiquitous from all kingdoms of life (3). Despite modest degrees of overall primary sequence homology, the basic structure fold of the α/β-hydrolases is extraordinarily conserved. However, diverse α/β-hydrolases have different substrate specificities, which contain a variety of enzymes, including esterases, lipases, proteases, dehalogenases, peroxidases, and epoxide hydrolases, and play important roles in life activities. Therefore, it is imperative to identify structural differences among various α/β-hydrolases. Esterases often show broad substrate spectrum and are widely used as biocatalysts for the synthesis of important materials in pharmaceutical and chemical industries (4). Although some esterase structures have been determined in recent years (5, 19, 30–34), no HSL esterase structure from a filamentous fungus has ever been reported. Here, we describe the structures of two HSL esterases from *R. miehei*, *Rm*EstA and *Rm*EstB, and elucidate the mechanism governing their different substrate specificities. The crystal structures of *Rm*EstA and *Rm*EstB allow us to address the molecular details of substrate binding and catalysis of the short-chain esters being different from other α/β-hydrolase superfamily esterases/lipases. Site-directed mutagenesis and structure-based rational design experiments can then be performed to search for enzymes with the improved catalytic efficiency and/or suitability for industrial applications.

The three-dimensional structures of both *Rm*EstA and *Rm*EstB exhibit the typical α/β-hydrolase fold with a core consisting of eight β-sheets surrounded by α-helices. The structures of both *Rm*EstA and *Rm*EstB are composed of two clearly distinguishable domains, a catalytic domain and a cap domain (Fig. 2), which are similar to those of most other esterases from the HSL family. However, several differences were found in the cap domain, for which α-helices α1 and α2 showed the highest *B*-factors (data not shown). The cap domain of HSL family esterases generally has a poorly conserved amino acid sequence but is structurally similar to other esterases/lipases. This domain makes an important contribution to several aspects of HSL enzyme function, including enzyme activity, substrate specificity, regioselectivity, thermophilicity, and thermostability (29). The α/β-hydrolase fold family members have a highly conserved nucleophile-His-acid catalytic triad, with Ser as the nucleophile and Asp or Glu as the acid (17, 35). The nucleophiles of *Rm*EstA and *Rm*EstB are Ser161 and Ser164, respectively, positioned in the conserved sequence Gly-X-Ser-X-Gly at a sharp turn connecting β5 and α6, similar to the esterase EstE1 (5).

**Fig. 5.**
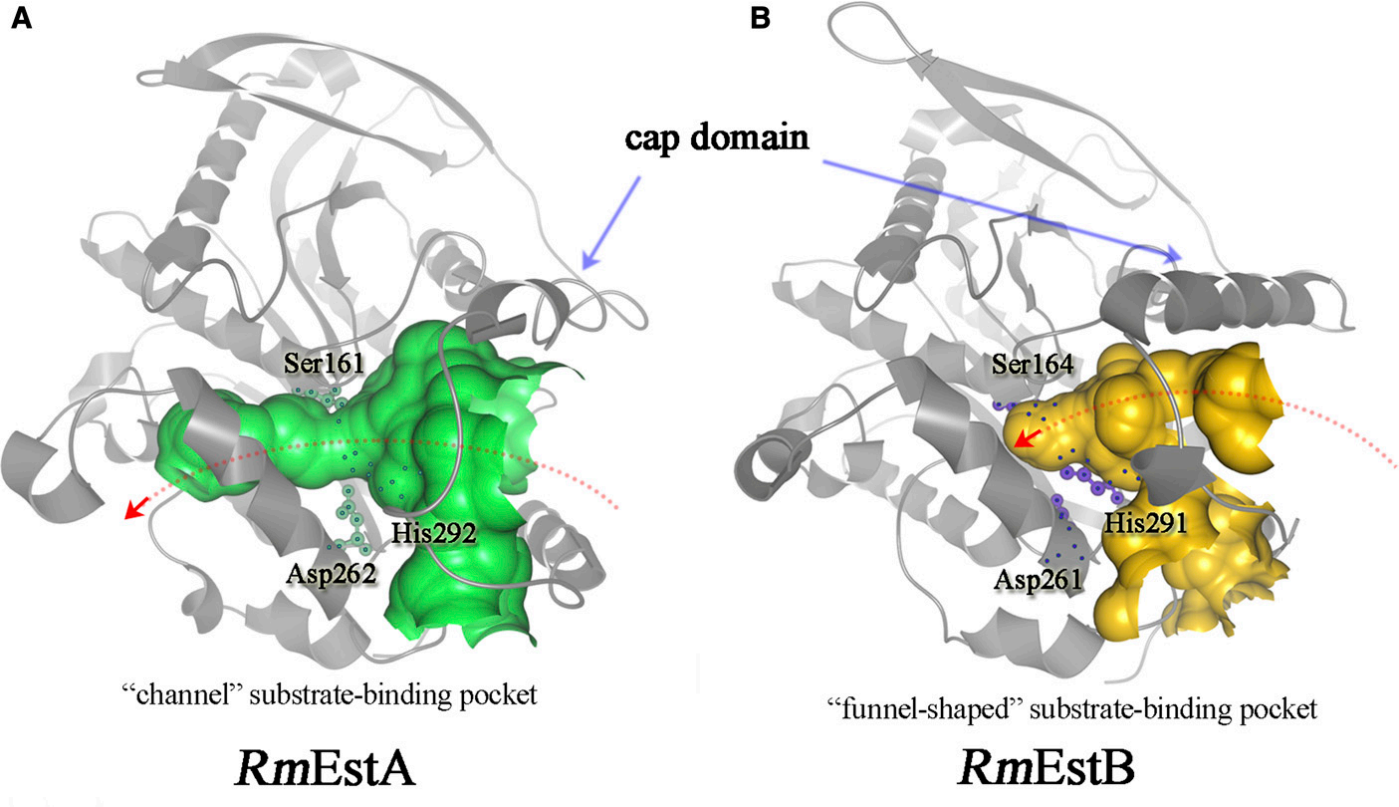
Visualization of substrate-binding pockets of *Rm*EstA and *Rm*EstB. To display their differences, Caver software analysis was carried out. The substrate-binding pockets of *Rm*EstA (A) and *Rm*EstB (B) are shown in green and brown, respectively. The residues of the catalytic triad are shown in dot and line models. The substrate-binding pocket of *Rm*EstA is shown as a curved tunnel going through the molecule. The substrate-binding pocket of *Rm*EstB is shown as a funnel-shaped groove.

Previous biochemical characterizations of *Rm*EstA and *Rm*EstB have indicated distinct substrate specificities for the two enzymes. *Rm*EstA can hydrolyze esters of longer carbon chain lengths (up to C16), with the highest activity observed for C6 (15), whereas *Rm*EstB favors the hydrolysis of esters with shorter carbon chain lengths, with highest activity observed for C2 (16). These properties differ from most other HSL family esterases, which show the highest activity for butyrate (C4) or caproate esters (C6) (36, 37). A structural comparison of *Rm*EstA and *Rm*EstB suggested that the differences in the substrate-binding pocket might make a marked contribution to their distinct substrate specificities. The substrate-binding pocket of *Rm*EstB is funnel-shaped with ∼11 Å from the protein surface to the catalytic residue Ser164, a distance that just fits the acyl chains of substrates with carbon chain lengths shorter than C4. Esters with acyl chain lengths longer than C4 therefore hardly bind to the substrate-binding site due to steric hindrance (**Fig. 5**). On the other hand, the substrate-binding pocket of *Rm*EstA is a curved tunnel which can accommodate ester substrates with long acyl chains (Fig. 5).

**Fig. 6.**
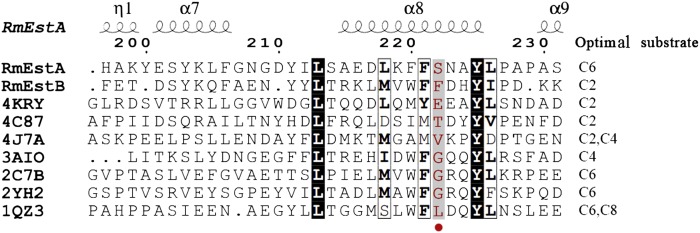
Partial multiple sequence alignment of selected esterases. Labeling with PDB codes: 4KRY, *E. coli* acetyl esterase; 4C87, *L. plantarum* esterase; 4J7A, metagenomic library esterase A; 3AIO, *S. tokodaii* esterase; 2C7B, metagenomic library esterase B; 2YH2, *P. calidifontis* esterase; and 1QZ3, *A. acidocaldarius* esterase. The key residue Phe222 of *Rm*EstB is labeled with a red dot in the amino acid sequence alignment. The optimal substrates of these esterases are listed, taken from previous studies (5, 30–34, 38).

The structural differences between the substrate-binding pockets of the two esterases suggest that several aromatic residues (Phe222 and Trp92) block the substrate-binding pocket in *Rm*EstB (Fig. 4B), which might contribute to the restriction in carbon chain lengths of the ester substrates. Phe222 and Trp92 are located in the center of the substrate-binding pocket, close together (4.1 Å). Aromatic residues in the substrate-binding pocket can transform the substrate specificity of esterase, as confirmed in the other previous study (18). To confirm the function of aromatic residues Trp92 and Phe222 in *Rm*EstB’s substrate specificity, two mutants, *Rm*EstB-F222A and *Rm*EstB-W92F, were created. The substrate specificities of mutant *Rm*EstB-F222A for *p*NPB (C4) and *p*NPH (C6) were enhanced by 1.65- and 1.4-fold, respectively (Table 2). The mutant *Rm*EstB-W92F showed similar substrate-specificity changes, with 1.33- and 1.11-fold enhanced specificity for *p*NPB (C4) and *p*NPH (C6), respectively (Table 2). The substrate specificities of the two *Rm*EstB mutants toward long-chain substrates also increased significantly compared with that of the wild-type *Rm*EstB (Table 2). Structural comparison and mutagenesis analysis in the present study indicated that the side chains of residues in the substrate-binding pocket create a steric hindrance, thereby potentially altering substrate specificity in the esterases; moreover, residue Phe222 played a vital role in *Rm*EstB’s variation in substrate specificity. The results of multiple sequence alignment analysis further confirmed this interference. Residues with large side chains were found at positions corresponding to position 222 of *Rm*EstB in esterases showing a preference for the hydrolysis of *p*NPA (C2), such as those from *Escherichia coli* [PDB code: 4KRY; corresponding residue: glutamate (30)], *Lactobacillus plantarum* [PDB code: 4C87; corresponding residue: threonine (31)], and metagenomic library [PDB code: 4J7A; corresponding residue: valine (34)] (**Fig. 6**). On the other hand, esterases with high activity toward *p*NPB (C4) or *p*NPH (C6) had relatively small side chain residues at position 222, such as those from *S. tokodaii* [PDB code: 3AIO; corresponding residue: glycine (32)], metagenomic library [PDB code: 2C7B; corresponding residue: glycine (5)], *P. calidifontis* [PDB code: 2YH2; corresponding residue: glycine (33)], and *A. acidocaldarius* [PDB code: 1QZ3; corresponding residue: leucine (38)] (Fig. 6).

## CONCLUSION

The three-dimensional structures of two HSL esterases from *R. miehei*, *Rm*EstA and *Rm*EstB, were determined. Both of the esterases were composed of a core catalytic domain and a cap domain, exhibiting the typical α/β-hydrolase fold. The side chains of residues in the substrate-binding pocket may create steric hindrance, thereby altering the substrate specificity of the esterases. The results in the present study may be helpful for the construction of new variants to improve substrate specificity of esterases and for further exploration of biotechnological applications.****
